# Label-Free Malignancy Phenotyping of Living Cancer Cells by High-Performance Surface-Enhanced Raman Spectroscopy Substrates

**DOI:** 10.3390/mi17040461

**Published:** 2026-04-09

**Authors:** Jiwon Yun, Hyeim Yu, Youngho Yun, Wonil Nam

**Affiliations:** 1 Department of Intelligent Robotics Engineering, Pukyong National University, Busan 48513, Republic of Korea; 202112150@pukyong.ac.kr (J.Y.); rhi0118@pukyong.ac.kr (H.Y.); 2 Department of Statistics, Virginia Polytechnic Institute and State University, Blacksburg, VA 24061, USA; yyun1@vt.edu; 3 Department of Electronic Engineering, Pukyong National University, Busan 48513, Republic of Korea

**Keywords:** surface-enhanced Raman spectroscopy, label-free, living cells, cancer, multivariate analysis

## Abstract

Surface-enhanced Raman spectroscopy (SERS) amplifies Raman scattering by placing molecules in the near-field of plasmonic nanostructures, enabling label-free molecular fingerprinting. While attractive for living cell phenotyping, many cellular SERS works rely on internalized colloidal nanoparticles, leading to variable uptake/localization, aggregation-driven hotspot fluctuations, and potential cellular perturbation. Here, we report a chip-like Au/SiO_2_ nanolaminate SERS substrate that supports direct culture and label-free measurements of living cells on spatially defined hotspots without nanoparticle uptake. The periodic nanolaminate forms dense nanogaps and is engineered for 785 nm excitation, providing uniform enhancement over a large, culture-compatible area with high hotspot uniformity. By engineering the cell–substrate nano–bio interface, the platform enables reproducible acquisition of intrinsic cellular vibrational fingerprints under physiological conditions without Raman tags. Using MCF-7 and MDA-MB-231 breast cancer cells, we collected hundreds of spectra per line, and MDA-MB-231 exhibited broader spectral variations, indicating greater heterogeneity. Principal component analysis and linear discriminant analysis achieved 99% classification accuracy for MCF-7 and MDA-MB-231, and bright-field imaging confirmed preserved adhesion and canonical morphologies. This chip-based, label-free living cell SERS platform enables scalable, nonperturbative phenotyping and may support rapid malignancy classification and treatment response screening across subtle cancer states.

## 1. Introduction

Surface-enhanced Raman spectroscopy (SERS) is a label-free vibrational spectroscopy technique that amplifies Raman signals from molecules located in the near-field of plasmonic nanostructures, enabling molecular fingerprinting with high sensitivity [1,2,3,4,5,6]. SERS has been applied broadly in chemical sensing, environmental monitoring, food safety, and biomedical analysis and diagnosis [7,8,9,10,11,12,13,14]. In biomedicine, SERS has been widely adopted for target biomarker detection, disease diagnostics, and bioimaging, using both direct label-free sensing and indirect strategies based on surface functionalization and Raman tags [15,16,17,18,19]. SERS has played a significant role in cellular and single-cell studies, offering practical advantages over fluorescence readouts, including narrow spectral bands, minimal photobleaching, and the ability to provide intrinsic chemical information from endogenous biomolecules [20,21,22,23,24].

For example, Spedalieri et al. used colloidal gold nanostars as intracellular SERS probes to analyze the cellular environment. They showed that nanostar morphology influences intracellular processing and the intracellular protein corona within endolysosomal compartments, enabling sensitive SERS readout of nano–bio interactions in cells [25]. Xu et al. proposed a label-free workflow for living cell identification using iodide-modified silver nanoparticles and ultrafast line-illumination Raman mapping. They achieved accurate classification of normal and cancer cells [26]. Zhang et al. developed a cell-permeable SERS nanoreactor by engineering gold nanoparticles to incorporate glucose oxidase and a Raman reporter, enabling transduction of intracellular glucose into a measurable SERS signal [27]. However, the majority of cellular SERS approaches still rely on functionalized colloidal nanoparticles as internal probes. This creates recurring limitations, such as heterogeneous cellular uptake and localization, potential cytotoxicity or perturbation of cellular physiology, and uncontrolled aggregation, which increase hotspot variability [28,29,30,31,32].

To overcome these issues, solid chip-like SERS substrates that can provide spatially defined hotspots while allowing cells to be directly cultured on plasmonic nanostructures have been developed, thereby reducing reliance on colloidal nanoparticle uptake and improving measurement stability. Direct label-free SERS enables cellular studies by interrogating the intrinsic Raman signatures of molecular ensembles within plasmonic hotspots, without chemical modification, yielding information-rich spectroscopic fingerprints from living biological systems. For example, Nikelshparg et al. developed a planar SERS substrate based on aggregated gold nanostars immobilized on a slide, enabling direct measurement of living cells on a chip. They showed that long nanostar tips can perforate and penetrate the cell membrane, enabling label-free SERS readout of intracellular molecular composition from living cells without relying on free-colloid internalization [33]. Plou et al. reported a plasmonic substrate comprising a superlattice of Au nanoparticles, providing dense and reproducible hotspots in a chip-like format. They demonstrated multiplex SERS detection of metabolic alterations in tumor extracellular media, leveraging the distinct vibrational fingerprints of metabolites without requiring nanoparticle uptake by cells [34]. We previously demonstrated refractive-index-insensitive SERS substrates for label-free Raman profiling and classification of living cells. The platform supported large-area substrates compatible with conventional cell culture and delivered high SERS performance with excellent hotspot uniformity while remaining insensitive to variations in the background refractive index (RI) [35]. While this approach achieved clear separation of two cell lines, the proof-of-concept study was limited to readily distinguishable classes (e.g., normal vs. cancer) rather than subtler phenotypic differences that are more relevant to real-world biological and clinical applications.

Here, we demonstrate label-free living cell SERS-based malignancy classification using breast cancer cell models with distinct aggressiveness (MCF-7 and MDA-MB-231) cultured directly on chip-type plasmonic substrates, establishing a measurement framework that does not require colloidal nanoparticle internalization. By leveraging a previous nanolaminate SERS platform, which enabled binary classification between cancer and normal cells, this work advances toward malignancy phenotyping by resolving subtle differences among closely related cancer cells. Specifically, we leverage a cell–substrate nano–bio interface engineered to present dense, spatially defined plasmonic hotspots accessible at the membrane-contact region, enabling reproducible acquisition of intrinsic cellular vibrational fingerprints. Because label-free cellular SERS is information-rich yet intrinsically high-dimensional and heterogeneous, we apply multivariate analysis, principal component analysis (PCA) combined with linear discriminant analysis (LDA), to capture subtle, distributed spectral differences beyond manual single-peak interpretation and to improve robustness against spectrum-to-spectrum variability arising from local hotspot sampling and cell–substrate coupling. Using this approach, we achieve high-accuracy discrimination between MCF-7 and MDA-MB-231 from hundreds of living cell spectra while preserving cell morphology on the substrate, thereby supporting both biocompatibility and classification performance in a chip-based format. These results highlight that designing the nano–bio interface in conjunction with spectral analysis enables practical label-free cellular SERS classification, providing a route toward more realistic phenotypic studies beyond highly separable classes.

## 2. Materials and Methods

SERS substrate fabrication: A nanowell-patterned polydimethylsiloxane composite stamp (period: 400 nm, diameter: 100 nm, and height: 150 nm) (SYLGARD^®^ 184 silicone elastomer, Dow Corning, Midland, MI, USA as replicated from a nanopillar-patterned silicon master using soft lithography. The stamp was then used to mold UV-curable polyurethane (PU) into nanopillar arrays on a flexible polyester film, followed by UV curing and thermal curing at 80 °C. Next, alternating Au/SiO_2_ multilayers consisting of four Au layers (30 nm each) and three SiO_2_ layers (6, 8, and 12 nm from the bottom to the top) were deposited by electron beam evaporation. Finally, the SiO_2_ layers were slightly etched in buffered oxide etchant (10:1) to expose the nanogap-based plasmonic hotspots.

Cell culture: MDA-MB-231 cells were obtained from Sigma-Aldrich (St. Louis, MO, USA) and grown in F12:DMEM containing 4 mM glutamine, 10% fetal bovine serum (FBS), and penicillin–streptomycin. MCF-7 cells were grown in EMEM with 10% FBS and 2 × L glutamine. Cells were cultured at 37 °C in a humidified atmosphere of 5% CO_2_ in air. Cells were then trypsinized and seeded on nanolaminate SERS substrates.

SERS measurement: We used a confocal Raman microscope (Thermo Fisher Scientific, Waltham, MA, USA) to acquire SERS spectra under 785 nm laser excitation. A 20× water immersion objective lens (NA = 0.5) was used with 5 mW of laser power and a 10 ms integration time. Calibration was performed based on the silicon peak.

Multivariate analysis: Cosmic ray removal, background subtraction, smoothing interpolation, and data truncation were carried out subsequently. Principal component analysis (PCA) and peak picking were performed in R software (version 4.5.1, R Core Team, Vienna, Austria) using the packages ChemoSpec and MALDIquant, respectively. Linear discriminant analysis (LDA) was performed using the R package MASS.

Finite-difference time-domain (FDTD) simulation: Optical simulations were performed using the FDTD solution in Ansys Lumerical (version 2020 R2.1, Vancouver, BC, Canada). A 2 nm mesh was used in all directions. The optical constants of gold were taken from Johnson and Christy. The Bloch boundary condition was used in the x and y directions with a periodicity of 400 nm, and the perfectly matched layer boundary condition was used in the z direction. The refractive indices of SiO_2_ and PU were set as 1.5 and 1.56, respectively.

## 3. Results

**Figure 1:**
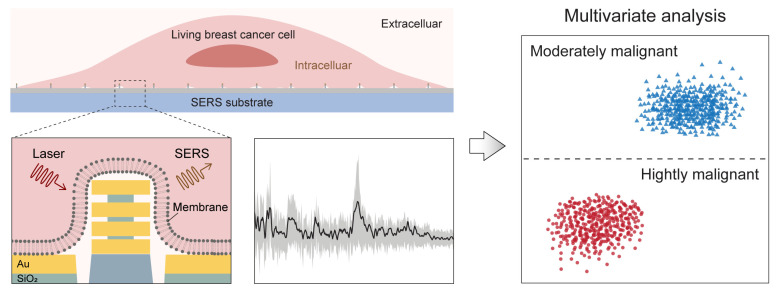
A schematic illustration of label-free SERS acquisition from living breast cancer cells for malignancy phenotyping. Protruding nanostructures can induce the membrane–hotspot interface, enabling the acquisition of the vibrational fingerprint information for multivariate analysis.

Figure 1 schematically illustrates the overall workflow and core concept of this study. Living breast cancer cells are cultured directly on a chip-like nanolaminate SERS substrate, where the cell membrane conforms to the surface topography and positions biomolecules within the near-field of densely distributed plasmonic hotspots at the cell membrane interface. Upon near-infrared laser excitation, this interface allows label-free SERS acquisition of the intrinsic vibrational fingerprints of living cells without introducing colloidal nanoparticles or Raman tags. Because the resulting cellular SERS spectra are information-rich yet heterogeneous and not readily interpreted by a few single peaks, we performed multivariate analysis to extract spectral differences and conducted malignancy-associated classification using principal component analysis (PCA) combined with linear discriminant analysis (LDA). In this work, we applied this approach to breast cancer models with distinct aggressiveness (MCF-7 and MDA-MB-231), demonstrating that chip-based label-free SERS is a practical tool for phenotypic discrimination by engineering a nano–bio interface.

**Figure 2:**
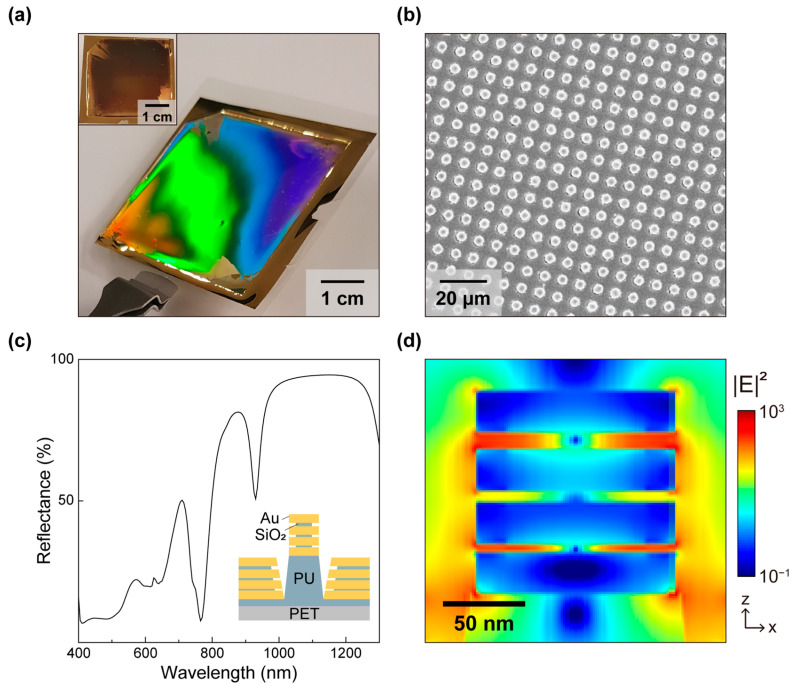
Characterization of nanolaminate SERS substrates. (**a**) Photograph of the nanolaminate SERS substrate. The inset shows the substrate from a different angle. (**b**) Top-view SEM image, (**c**) FDTD-calculated reflectance spectrum of the nanolaminate structure, and (**d**) spatial distribution map of |E|^2^ at 785 nm.

Figure 2a shows a representative optical photograph of the nanolaminate SERS substrate. Briefly, nanolaminate SERS substrates were fabricated by combining soft lithography, nanoimprint lithography, and physical vapor deposition to form periodic polymeric nanostructures, followed by conformal deposition of alternating metal–dielectric layers (Au-SiO_2_) to create nanogap-rich nanolaminate features. Detailed fabrication processes are described elsewhere [35]. The vivid diffraction observed over the patterned area qualitatively indicates periodic and highly uniform nanostructures, while the inset shows a dark appearance without diffraction. Figure 2b presents a scanning electron microscope (SEM) image confirming a highly periodic array of nanolaminate features with uniform pitch and geometry, consistent with wafer-scale replication by nanoimprinting. Figure 2c shows the finite-difference time-domain (FDTD)-calculated reflectance spectrum of the nanolaminate substrate from 400 to 1300 nm, together with a schematic cross-section of the multilayer stack. As gold exhibits strong interband transitions at ~520 nm [36], which increase optical loss and degrade the plasmonic response in the visible band, we designed the multilayer thicknesses to support resonant behavior in the near-infrared band around 785 nm. In addition, a multiresonant feature around 860 nm overlaps with Stokes-shifted Raman bands from biomolecules, thereby improving overall collection efficiency across the fingerprint window. Figure 2d displays the calculated near-field distribution |E|^2^ at 785 nm, revealing strong field localization in the nanoscale gaps formed within the nanolaminate structures along the sidewalls of the metallic structures that are physically accessible when the cell membrane conforms to the topography at the cell–substrate interface. The calculated maximum |E|^2^ value is 3.5 × 10^3^, corresponding to SERS enhancement on the order of 10^7^ under the commonly used |E|^4^ approximation [37]. This value is consistent with the measured SERS enhancement factor of about 10^7^ [30]. Consistent with this design, the substrate has shown high uniformity along with reproducible SERS responses across multiple measurements [38].

**Figure 3:**
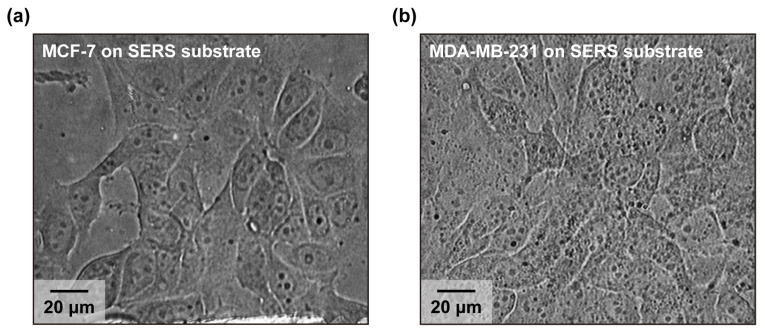
Bright-field images of living breast cancer cells: (**a**) MCF-7 and (**b**) MDA-MB-231.

Figure 3 shows bright-field images of living breast cancer cells on the nanolaminate SERS substrates. Cells were directly cultured onto SERS substrates without surface functionalization. We selected MCF-7 and MDA-MB-231 as benchmark breast cancer models that span distinct molecular subtypes and invasive phenotypes, enabling the evaluation of label-free living cell SERS measurements across a biologically meaningful range of malignancy levels. MCF-7 is an ER/PR-positive, luminal A-type line that is commonly treated as a model of lower metastatic and less aggressive breast cancer biology [39,40,41]. In contrast, MDA-MB-231 is a triple-negative line widely used as a model of high aggressiveness and invasiveness, with features associated with a mesenchymal-like phenotype [42,43]. This pair is frequently used to compare epithelial-like and mesenchymal-like behaviors and the degree of aggressiveness. Bright-field images show that both MCF-7 and MDA-MB-231 cells remain well adhered and spread on the SERS substrates, with no obvious widespread cell rounding, membrane blebbing, or surface detachment, which are commonly used morphological indicators of acute cell death and cytotoxic stress in adherent cultures [44,45]. MCF-7 (Figure 3a) cells display a polygonal epithelial-like morphology with cohesive colony formation, whereas MDA-MB-231 (Figure 3b) cells show a more elongated, spindle-shaped morphology with reduced cell–cell cohesion and more prominent protrusions. These features are consistent with the comparatively less invasive epithelial phenotype of MCF-7 and the more invasive, mesenchymal-like phenotype of MDA-MB-231 [43,46].

**Figure 4:**
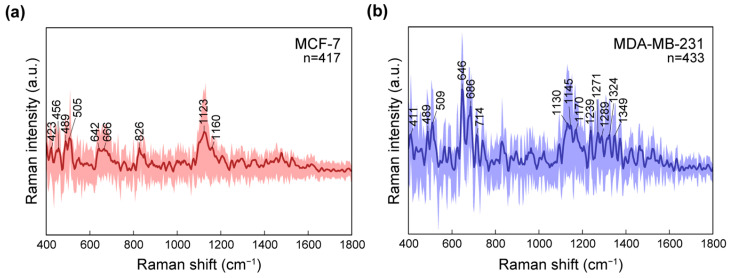
Representative average SERS spectra of living breast cancer cells with the 5th and 95th percentile envelopes and biomolecular characteristic peaks: (**a**) MCF-7 and (**b**) MDA-MB-231. The numbers of spectra are 417 (MCF-7) and 433 (MDA-MB-231).

We performed label-free SERS measurements on living breast cancer cells (MCF-7 and MDA-MB-231) cultured directly on SERS substrates. For each sample, spectra were acquired over a 100 μm × 100 μm mapping area using a 100 μm × 100 μm grid. After pre-processing and quality control, 417 spectra for MCF-7 and 433 spectra for MDA-MB-231 cells were retained for subsequent analysis. Figure 4a,b shows the average SERS spectra of MCF-7 and MDA-MB-231, respectively (solid line), and the shaded bands denote the 5th–95th percentile across the spectra. Notably, the MDA-MB-231 spectra exhibit a broader percentile range than those of MCF-7, indicating greater spectral heterogeneity under identical measurement conditions. This increased heterogeneity is consistent with the more aggressive, mesenchymal-like phenotype of MDA-MB-231 relative to the more epithelial-like MCF-7 and may reflect a combination of intrinsic biochemical diversity. Despite this variability, both cell lines show reproducible spectral features in the fingerprint region.

The band at 505 cm^−1^, assigned to disulfide (S–S) vibrations in proteins, showed slightly higher intensity in MDA-MB-231 cells than in MCF-7 cells. Disulfide bonds are common structural features in membrane-associated and extracellular proteins, and variations in their SERS intensity may reflect differences in protein composition or structural organization at the cell surface. The increased signal observed in MDA-MB-231 may therefore be associated with altered protein organization or extracellular matrix-related remodeling in the more aggressive phenotype [47]. The 646 cm^−1^ band, attributed to tyrosine-related protein vibrations, is also relatively enhanced in MDA-MB-231. Tyrosine residues are frequently present in membrane proteins and signaling-related molecules, and changes in their relative intensity may indicate differences in membrane protein abundance or the local molecular environment. This enhancement may reflect variations in membrane-associated protein composition between the two cell types [48,49]. In addition, the 1050–1500 cm^−1^ spectral region, which includes multiple bands associated with CH_2_ deformation and unsaturated lipid vibrations, exhibited higher overall intensity in MDA-MB-231 cells compared to MCF-7 cells. This region is commonly linked to membrane phospholipids and fatty acid-related molecular structures in cellular Raman spectroscopy. The increased signal across this lipid-associated region may therefore suggest differences in membrane lipid composition or organization, potentially reflecting the altered metabolic and structural characteristics of the more invasive cell line [50,51].

Manual peak-by-peak interpretation is useful for assigning dominant biomolecular bands, but it is often insufficient for robust cell-state discrimination in label-free cellular SERS for several reasons. First, cellular spectra are high-dimensional and highly congested; therefore, many bands overlap and represent mixed contributions from proteins, lipids, nucleic acids, and metabolites. Diagnostic differences are rarely confined to a single isolated peak. Second, absolute and even relative peak intensities may vary due to hotspot sampling effects and cell–substrate contact heterogeneity, making single-peak metrics sensitive to measurement variability and outliers. Third, phenotypic differences between cell lines typically manifest as subtle, distributed changes across multiple wavenumbers, which are difficult to capture with a limited set of hand-picked peaks. Accordingly, we applied multivariate analysis to leverage the full spectral fingerprint and extract the dominant variance structure in an unbiased manner.

**Figure 5:**
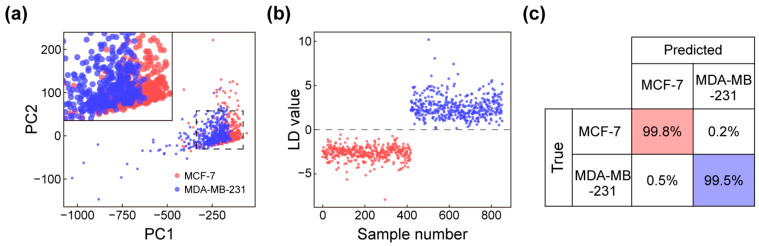
Multivariate analysis of living breast cancer cells. (**a**) PCA score plot of two breast cancer cell types. (**b**) PCA-LDA score plot comparing groups with different malignancy levels. (**c**) Confusion matrix for PCA-LDA-based classification of the two malignancy groups.

To evaluate whether label-free SERS fingerprints obtained from cells cultured on the SERS substrate are sufficiently robust for phenotypic discrimination, we performed multivariate analysis on spectra acquired from MCF-7 and MDA-MB-231 cells. PCA provides an unsupervised dimensionality reduction method that summarizes correlated spectral variations into a small number of orthogonal components. As PCA is an unsupervised method that does not explicitly maximize interclass separation, the extracted PC scores were used as input features for LDA. In this study, 24 principal components were retained, accounting for 95% of the total spectral variance. LDA then uses these reduced features to identify a projection that maximizes interclass separation relative to intraclass variance, yielding a quantitative classifier that is less dependent on any single Raman band and more robust to spectrum-to-spectrum intensity fluctuations. Unsupervised PCA provides an overview of spectral variance and reveals two dominant clusters corresponding to the two cell lines in the PC1-PC2 space (Figure 5a). However, a subset of points shows partial overlap (inset), consistent with expected biological heterogeneity and local variability at the cell–substrate interface.

We then applied LDA to PCA-reduced features (PCA-LDA) to maximize interclass separation, yielding LD scores that are clearly divided (Figure 5b). To rigorously estimate classification performance and minimize overfitting, we employed leave-one-out cross-validation (LOOCV), in which a single spectrum was held out as the test sample while the PCA-LDA model was trained on all remaining spectra. This procedure was repeated iteratively until each spectrum had been used once as the test case. As shown in Figure 5c, the resulting classifier achieves an accuracy of 99.8% for MCF-7 and an accuracy of 99.5% for MDA-MB-231, demonstrating that label-free SERS measurements retain sufficient molecular fingerprint information to distinguish two breast cancer cell models with distinct phenotypes.

## 4. Conclusions

In this work, we demonstrated label-free malignancy phenotyping of living breast cancer cells cultured directly on chip-type, nanolaminate SERS substrates, thereby avoiding the need for colloidal nanoparticle uptake. The nanolaminate structure provides large-area, highly uniform hotspots and is optically engineered to support strong near-field enhancement at 785 nm, enabling reproducible acquisition of intrinsic cellular vibrational fingerprints while preserving canonical cell morphologies on the substrates. Using hundreds of living cell spectra per class, we observed clear population-level spectral differences, with greater heterogeneity in the more aggressive phenotype. We achieved high classification accuracy between MCF-7 and MDA-MB-231 via PCA-LDA, supporting chip-based SERS as a practical platform for label-free cellular classification.

Several opportunities remain to advance this platform toward realistic biological and translational use. First, beyond binary separation, the approach should be extended to subtler phenotypic classification, including intermediate states, drug-induced cases, and clinically relevant intraclass variability, by expanding to multiple cell lines and patient-derived models. Second, to ensure robustness and generalizability, future studies should incorporate independent external test sets and explicitly quantify batch effects, for example, substrate-to-substrate, day-to-day, and operator-to-operator variability, supported by standardized protocols. Finally, integration with higher-throughput acquisition will help connect spectral classification to underlying biochemical mechanisms and move chip-based label-free cellular SERS toward practical phenotyping workflows. Overall, this work supports substrate-based living cell SERS as a route toward scalable, label-free phenotypic screening in cancer biology and related applications.

## Data Availability

The data presented in this study are available upon reasonable request from the corresponding author.

## Abbreviations

The following abbreviations are used in this manuscript:

SERS	Surface-enhanced Raman spectroscopy
RI	Background refractive index
PCA	Principal component analysis
LDA	Linear discriminant analysis
PU	Polyurethane
FBS	Fetal bovine serum
SEM	Scanning electron microscope
FDTD	Finite-difference time-domain
